# First evidence of biogenic habitat from tubeworms providing a near-absolute habitat requirement for high-intertidal *Ulva* macroalgae

**DOI:** 10.1371/journal.pone.0176952

**Published:** 2017-05-03

**Authors:** Kiran Liversage

**Affiliations:** Department of Environment, Water and Natural Resources, Government of South Australia, Mount Gambier, South Australia, Australia; University of Waikato, NEW ZEALAND

## Abstract

Disturbances in ecological systems can cause new resources to become available and can free the resources held by strongly competitive species. In intertidal boulder fields, wave-action causes disturbance by overturning boulders and freeing space for re-colonisation. In this study, mensurative experiments showed that boulder disturbance may also cause new biogenic-habitat resources to become available, if pre-disturbance boulders originally had tubeworm encrustations on their undersides. On the high-shore of a South Australian rocky coast, a small proportion of boulders had extensive encrustations of serpulid and spirorbid worm-tubes on their uppersides, and were likely to have recently been overturned, as spirorbid tubeworms are almost always only underneath boulders while living. *Ulva* macroalgae was absent from all boulders, except those with worm-tubes, where up to 61% *Ulva* cover was observed. Many boulders with tubes did not, however, have much algae, and this was likely caused by grazing. While limpets were seldom observed attached to tube encrustations, snails such as *Nerita atramentosa* and *Bembicium nanum* were equally abundant on and off tubes. *N*. *atramentosa* was likely the main grazer, as its densities were negatively correlated with *Ulva* cover. The mechanism causing association of *Ulva* and worm-tubes is unknown, but may be related to retention of moisture or algal spores within the complex topography of the tubes. Alternatively, some tubes may still have been living and providing nutrients for *Ulva* from excretory products. This study takes the first step towards understanding a very distinct habitat requirement which allows an important alga to persist in the hostile environment of the rocky-intertidal high shore.

## Introduction

The resources for species that are made available following disturbances are of ubiquitous importance in ecological systems [1]. Resources that become available following disturbance include light [2], water [3, 4] space on a substratum [5], and nutrients [6, 7]. Disturbance is generally variable across a land or sea-scape, resulting in “patchwork” mosaics of disturbed and undisturbed areas, with species adapted for early successional stages abundant in recently disturbed patches while strongly competitive species dominate patches in later stages [8, 9]. Models of succession suggest that early colonising species can affect later colonisers variably, via tolerance, inhibition or facilitation [10]. In recent years, the importance of the facilitation model has been emphasised [11], as levels of habitat complexity [12] and species diversity [13] have been found to be driven largely by this process. It is considered especially relevant to current ecological theory for more research on how facilitation can broaden species niches and affect biotic and abiotic heterogeneity [14].

Much of our understanding of disturbance [5, 15] and models of succession [16–18] has originated from research on rocky shore systems. Wave-action can directly cause mortality and release of resources [19, 20], or it can shift unstable substrata and cause mortality indirectly [21, 22]. When space for colonisation on rocky shore substrata is limited, disturbance can be a predominant structuring force, if it allows release of that resource [5]. Disturbance to canopy forming intertidal algae can likewise free light resources for turf-forming species [23]. Seldom are other types of resources that can be released by disturbance considered in rocky intertidal ecology. The present study investigates another type of disturbance-related resource, biogenic habitat, with the general aim of highlighting how other diverse forms of resources can be provided by disturbance, not just space and light.

Ecological processes such as competition, predation and grazing can leave distinct patterns, allowing inference of the spatiotemporal extent of those processes by observing indicative patterns [24–26]. On intertidal reefs consisting of unstable substrata such as boulders, the patterns caused by disturbance are well known [21, 27, 28]. In this study, patterns were observed of disturbance, and the provision of new ecological resources via disturbance, on a rocky shore in South Australia characterised by large boulders. The undersides of intertidal boulders are often encrusted with large densities of worm-tubes, mostly serpulids and spirorbids [29, 30]. Covers of these tubes can sometimes reach nearly 100% [31], and they are exposed on the uppersides when wave-action causes the boulder to overturn [29]. The tubes can persist for several weeks at least [32] and the empty tubes can provide habitat for other species (e.g. isopods [33]). Similarly, the structure provided by subtidal serpulid tube aggregations can harbour large species diversities [34]. If serpulid and spirorbid tubes on wave-overturned intertidal boulders similarly harbour large species diversities, then this disturbance-type may be initiating a process of successional facilitation, with associated system-wide implications [12], which can be preliminarily understood by analysis of the naturally-occurring patterns.

This study investigated patterns on the high-shore of the rocky intertidal in South Australia, where calcareous tubes of serpulid (*Galeolaria caespitose*) and spirorbid polycheates were abundant on top of a small proportion of boulders. Spirorbids in particular are known to be associated only with substrata on undersides and edges of boulders while living [32, 35], so boulders with large covers of tubes of these worms on their tops were presumed to have been recently overturned [29]. Preliminary observations suggested that these boulders harbour populations of *Ulva* spp. that were not observed in other habitats. In this study, a mensurative experiment (sensu [36]) was done to test the hypothesis that the percentage cover of *Ulva* spp. is greater on boulders with serpulid and spirorbid (together, serpulimorph [37]) tubes compared to co-occurring boulders without tubes.

This experiment was done in the context of a system characterised by high densities of benthic grazers [38, 39] which use *Ulva* spp. as an important food source [9, 40]. To assess possible effects of these grazers, the hypothesis was tested that grazer assemblages would differ on boulders with vs without serpulimorph tubes. Also, the related hypothesis was tested that grazer assemblages would differ between areas encrusted with tubes vs areas free from tubes, on individual boulders. Finally, the specific grazers that were causing patterns were investigated by testing correlations of separate species with cover of *Ulva* spp.

## Materials and methods

**Fig 1:**
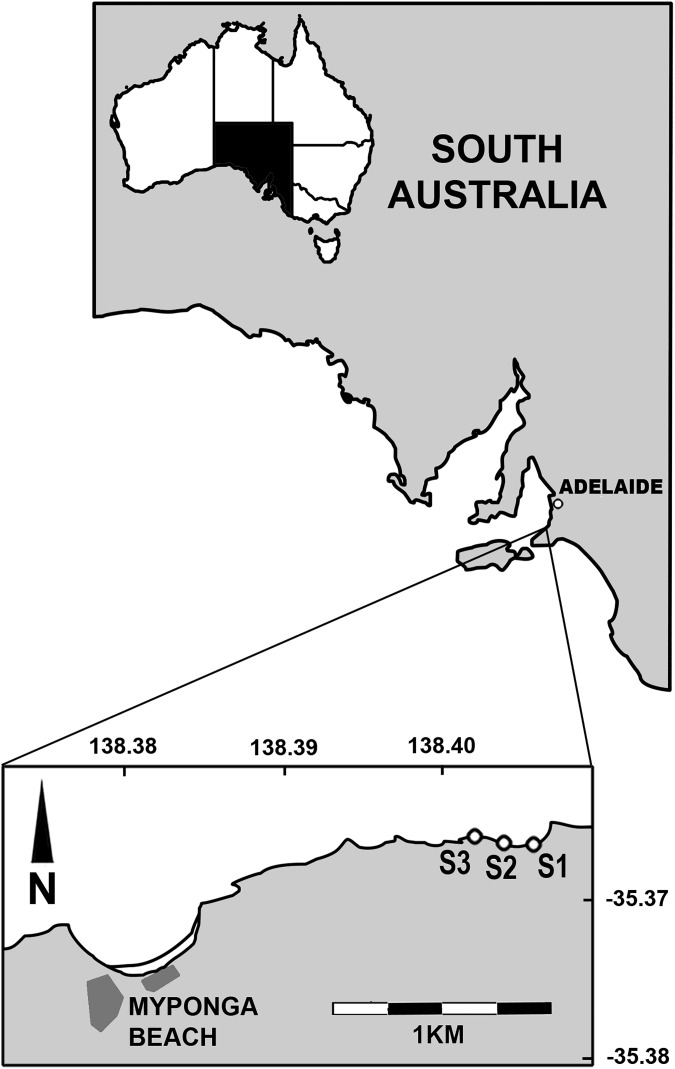
Map of the Myponga Beach coastline in South Australia, showing the three sites (S1, S2 and S3) where boulders were sampled.

The experiment was done on the rocky shoreline 1.9km east of Myponga Beach, on the Fleurieu Peninsula, South Australia (Fig 1). The Myponga Beach coastline comprises active cliffs above shore platforms strewn with large irregular-shaped boulders. The relative hardness of the siltstone, sandstone and quartzite rock types at Myponga Beach is a factor that contributes to these boulders being an ideal habitat for a range of benthic species [38, 41].

**Fig 2:**
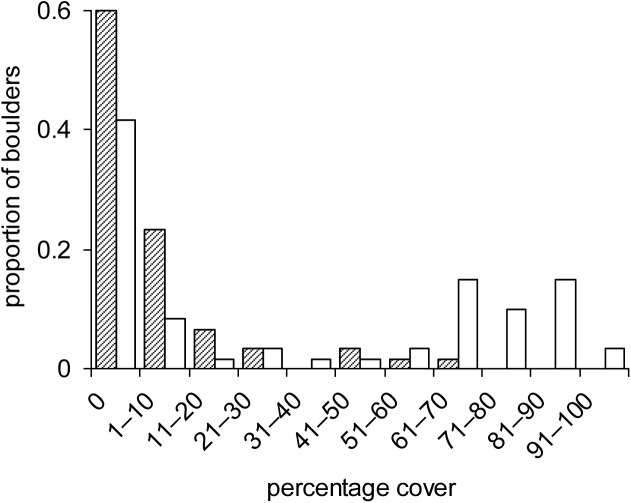
Frequency distribution of percentage covers of algae (filled bars), and tubes of serpulimorph polychaetes (white bars) across the sampled boulders.

Observations were made in August 2016 at three sites evenly spaced along 300m of the high-shore (Fig 1). On their uppersides, boulders generally had either very few serpulimorph tubes, or a large cover (13–95%, mean = 67%; Fig 2). At each site ten boulders were photographed with serpulimorph tubes and ten without, with scales bars included in the photographs. All boulders were haphazardly selected (see [42]) and intermixed within the same tidal height. Sampled boulders were generally separated from each other by about one meter.

The photographs were imported into the programme SketchUp v8 (www.sketchup.com) and adjusted to correct size using the scale bars. The programme was used to calculate two-dimensional boulder areas and areas covered with serpulimorph tubes and *Ulva* spp. The size of boulders can affect attached biota (e.g. [28, 43, 44]), so ANOVA, using WinGMAV5 (EICC, The University of Sydney), was used to check that boulder size (here measured as two-dimensional boulder area) was similar between boulders with vs without tubes. The boulders were mostly flat but also slightly ovoid-shaped, so the area of the visible boulder surface is slightly underestimated when measured from a two-dimensional photograph [32]. Boulders in the different treatments, however, were similar in size and shape, so any difference between actual area and two-dimensional area caused by boulder curvature was similar among treatments. All mobile species found were grazers; their densities were calculated by dividing numbers by boulder area. After arcsine transformation [45], percentages of *Ulva* spp. were compared between boulders with and without serpulimorph tubes (fixed factor) from the three sites (random factor). All data had heterogenous variances that could not be made homogenous using transformations, so analyses were done using univariate PERMANOVA in PRIMER v6 which is robust to departures from this assumption [46]. Euclidean distance matrices were used that were calculated from single variables, which give the same *F*-statistic as ANOVA [47]. PERMANOVA allows covariates to be included to yield an ANCOVA model [48]; here the covariate was grazer density (all species combined), with interactions included so the model can allow different slopes of the continuous variable for different levels of the categorical factors.

Grazer assemblages from the three sites were compared between boulders with vs without serpulimorph tubes using PERMANOVA based on Bray-Curtis similarities [48]. Processes affecting the use of these boulders by mobile species may or may not be dependent on their densities, so separate analyses were done for grazer densities and grazer numbers per boulder. A dummy value of one was added in both analyses due to sparsity of values among samples [48]. The abovementioned comparisons were *between* boulders with vs without tubes; another analysis was done to compare assemblages on areas *within* surfaces of boulders. This analysis only included boulders on which tubes occurred, and made comparisons between the areas on tubes vs the areas where no tubes occurred (i.e. bare rock). Each boulder in this analysis was randomly chosen to have measurements taken either on the tubes or off them, to ensure replicates were independent [45]. Across all these boulders, the mean area on tubes was 1292 cm^2^ and off tubes was 582 cm^2^. Counts of animals were divided by the respective area sampled for each boulder to standardise across differing areas, and this analysis was only done using these densities and not unstandardised counts. PERMANOVA pairwise tests showed where differences occurred among treatments, and patterns were visualised on nMDS plots. Patterns of individual species differences were determined using SIMPER (Similarity Percentages), which calculates the percentage contribution of each species to dissimilarity among treatments [48].

To test relationships of separate grazer species with cover of *Ulva* while including effects of the “Site” factor, correlations were determined between *Ulva* cover and grazer abundances (densities and numbers per boulder) using permutational ANCOVA, with Site as a random categorical factor. Analyses were done separately for each grazer species with a sufficiently great abundance (≥300 individuals.m^-2^). All analyses had 9999 permutations and used Type III sums of squares. When interaction terms that included a random factor were non-significant (*P* > 0.25) they were eliminated to increase the power of tests for relevant null-hypotheses [45].

## Results

**Fig 3:**
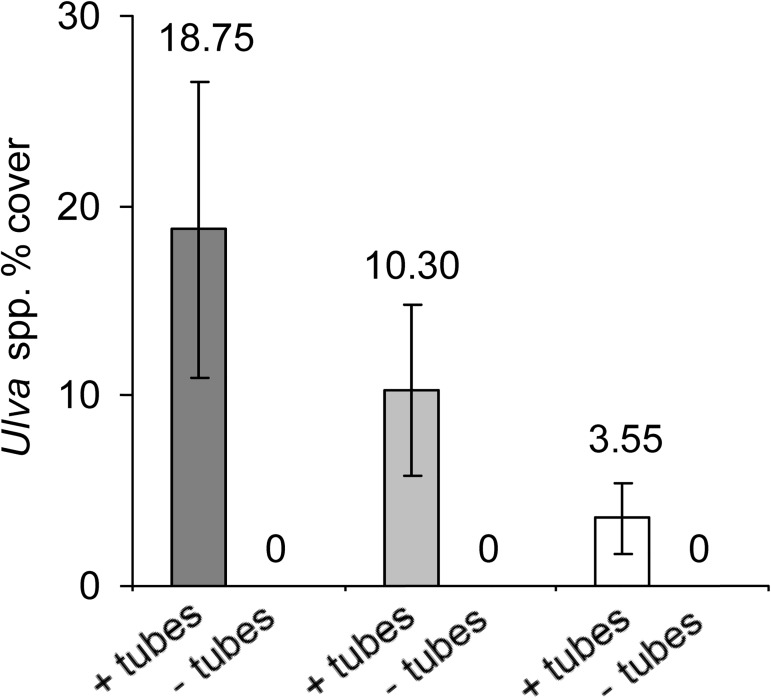
*Ulva* algae is present on boulders with worm-tubes and absent from boulders without. Mean (±SE) percentage cover of *Ulva* spp. on boulders with and without calcareous tubes of serpulimorph polychaetes. Dark grey bars are from Site 1, light grey from Site 2, and white from Site 3; *n* = 10.

Most boulders did not have any serpulimorph tubes or had only small covers, but another group of boulders had covers from 63–97% (Fig 2). Similarly, most boulders had no *Ulva*, while a smaller proportion had larger covers, in one case reaching 61% (Fig 2). At all three sites, *Ulva* spp. was never observed on any boulders that did not have tubes (Fig 3), leading to a significant difference between the boulder types (Table 1). The large amounts of variance heterogeneity among treatments may have contributed to this significant outcome, but a biological effect is highly likely as the dependent variable was completely without values in one set of treatments (Fig 3). When counts of all grazing species were combined, there was no evidence that their densities affected this pattern (Table 1). Boulders with and without tubes were of similar size (ANOVA, *F*_(1,58)_ = 0.55, *P* > 0.25), averaging 1879 cm^2^ in two-dimensional area.

**Table 1:** Amounts of *Ulva* algae differ significantly between boulders with vs without worm-tubes.

source	df	MS	*F*	
Grazer covariate (Gr)	1	126.44	1.05	
Site (Si)	2	199.29	1.66	
Tube presence (Tu)	1	1111.60	9.26	**
Gr x Si	2	–		
Gr x Tu	1	3.65	0.03	
Si x Tu	2	–		
Gr x Si x Tu	2	–		
Residual	54	120.03		

Analysis was done with PERMANOVA using Euclidean distances [48]. Comparisons were of percentage cover of *Ulva* spp. on boulders with and without the presence of serpulimorph tubes from three random sites and with the density of all grazers as covariate. Eliminated interaction terms (*P* > 0.25) are denoted by “–”; *n* = 10.

**P < 0.01.

**Fig 4:**
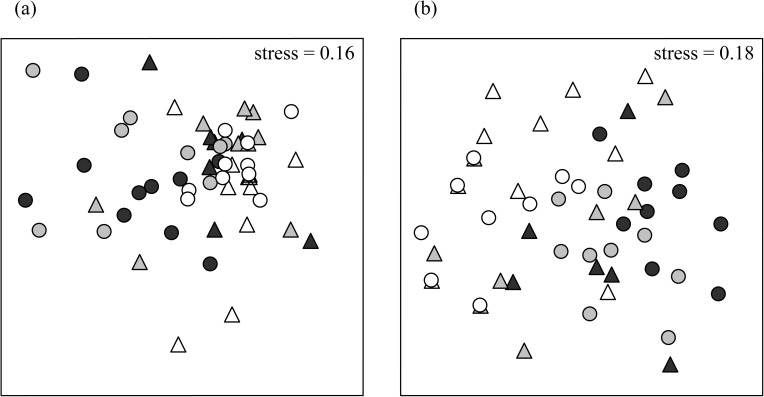
Associations of grazer assemblages with worm-tubes vary according to random sites. nMDS plots of (a) densities, and (b) numbers per boulder, of mobile assemblages (grazers) on boulders with serpulimorph tubes (triangle) and without (circle). Dark grey symbols are from Site 1, light grey from Site 2, and white from Site 3. Results from PERMANOVA pairwise tests are shown under the plots; *n* = 10.

**Fig 5:**
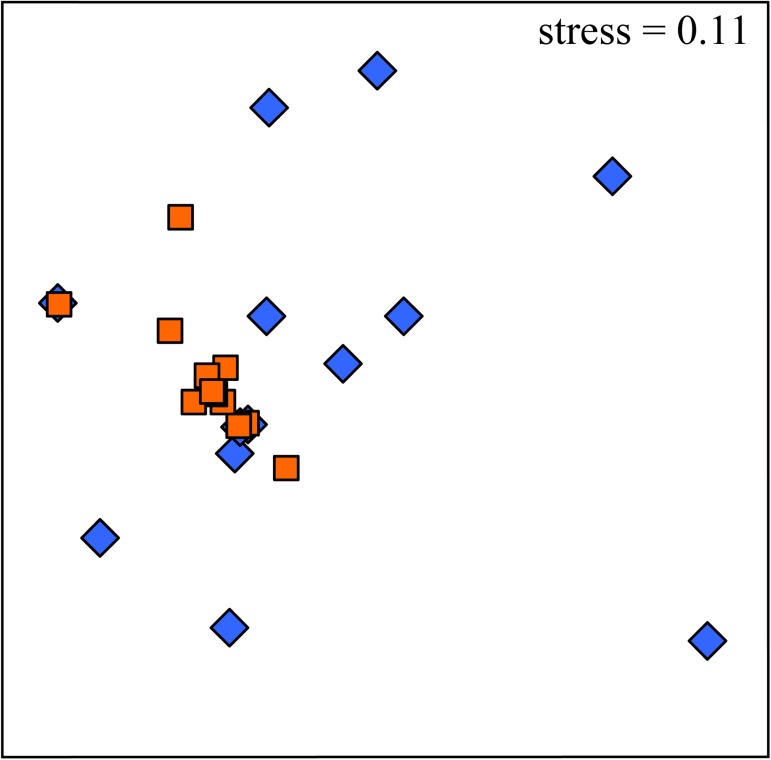
Grazer assemblages differ between areas on vs off worm-tubes. nMDS plot of densities of mobile assemblages (grazers) on areas of boulders on top of serpulimorph tube encrustations (orange squares) and on areas of boulders off encrustations (blue diamonds). Data shown are pooled from three random sites; *n* = 5.

Five mobile species were observed; *Nerita atramentosa*, *Cellana tramoserica*, *Bembicium nanum*, *Siphonaria denticulata* and *Patelloida latistrigata*. Limpets of the genus *Notoacmea* were also found but were not able to be identified to species level, so the taxon was included as *Notoacmea* spp. Assemblages were not consistently different between boulders with vs without serpulimorph tubes (Table 2, Fig 4A and 4B). On boulders with serpulimorph tubes, assemblages did differ consistently between substrata on vs off the tubes (Table 2, Fig 5). SIMPER showed that no one species mostly caused this pattern, but differences in abundance were strong especially for *C*. *tramoserica* and *S*. *denticulata*, which had large abundances off tubes but were seldom found on them (Table 3).

**Table 2:** Grazer assemblages differ significantly between areas on vs off worm-tubes.

		density				no. per boulder		
		df	MS	*F*		MS	*F*	
boulders with vs without tubes	Site (Si)	2	678.05	3.27	**	6171.5	3.51	***
	Tube presence (Tu)	1	1297.50	2.11		6184.1	1.35	
	Si x Tu	2	614.53	2.96	**	45.90.1	2.61	**
	Residual	54	207.40			1759.4		
areas of boulders on vs off tubes	Site (Si)	2	408.28	1.17				
	Tube presence (Tu)	1	1212.40	3.48	**			
	Si x Tu	2	–					
	Residual	26	348.23					

Analyses were done with PERMANOVA using Bray-Curtis similarities [48] comparing assemblages of grazers on boulders with and without the presence of serpulimorph tubes (*n* = 10), and on areas of boulders on and off tubes (*n* = 5) from three random sites. Eliminated interaction terms (*P* > 0.25) are denoted by “–”; *P < 0.05; **P < 0.01; ***P < 0.001.

**Table 3:** SIMPER analysis results.

species	density.m2 off tubes	density.m2 on tubes	dissimilarity: standard deviation ratio	% contribution to dissimilarity
*Nerita atramentosa*	12	12	0.89	31.04
*Cellana tramoserica*	22	0	0.75	23.25
*Bembicium nanum*	6	6	0.65	17.37
*Siphonaria denticulata*	17	3	0.58	17.24
*Patelloida latistrigata*	5	0	0.32	7.91
*Notoacmea* spp.	5	0	0.27	3.19

Contributions of species to measures of dissimilarity for grazer assemblages on areas of boulders overlying encrustations of serpulimorph tubes, and areas off the tubes.

**Fig 6:**
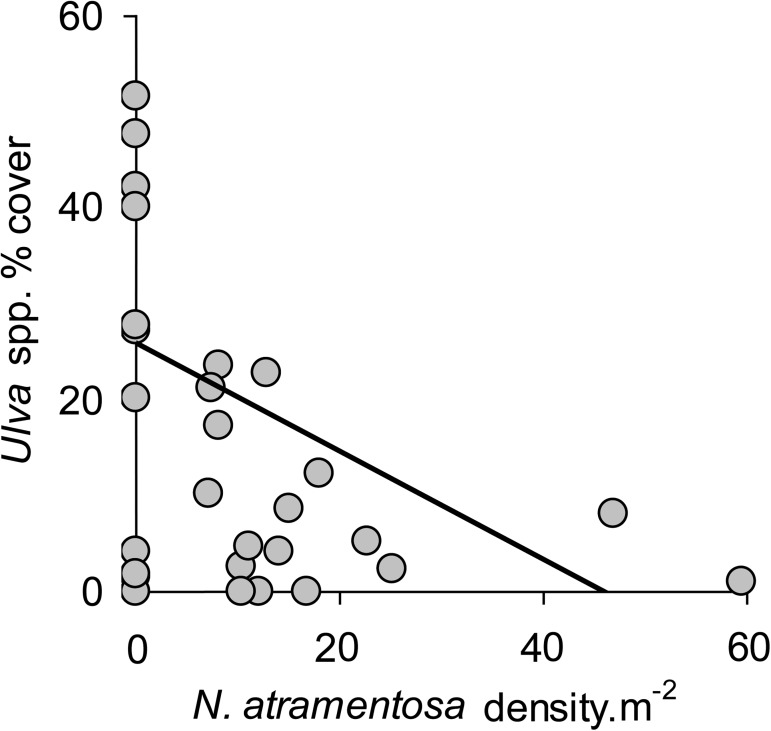
Density of the grazer *Nerita atramentosa* is positively correlated with *Ulva* algae cover. Correlation between density of the grazer *N*. *atramentosa* and cover of *Ulva* (arcsine transformed; [49]) on boulders which had serpulimorph tubes. Linear regression was used to visually represent the result from the permutational ANCOVA. Data were pooled from three random sites; *n* = 10.

When abundance of the four most common grazers were correlated with cover of *Ulva*, there was evidence that one species (*N*. *atramentosa*) contributed to variability of *Ulva*. Concerning *N*. *atramentosa* densities, there was a negative correlation between the grazer and the alga (Table 4, Fig 6), although this relationship was not observed when numbers of *N*. *atramentosa* per boulder were considered (Table 4). No correlations were observed for other grazers (Table 4).

**Table 4:** Density of the grazer *Nerita atramentosa* is significantly correlated with cover of *Ulva* algae.

			*N*. *atramentosa*				*C*. *tramoserica*		*B*. *nanum*		*S*. *denticulata*	
		df	MS	*F*			MS	*F*	MS	*F*	MS	*F*
density per boulder	Site (Si)	2	413.41	2.01			415.28	1.75	393.77	1.65	314.50	1.46
	Abundance (Ab)	1	868.72	4.23		*	47.30	0.20	15.45	0.06	535.51	2.49
	Si x Ab	2	–				–		–		341.35	1.59
	Residual	26	205.22				236.81		238.04		215.04	
no. per boulder	Site (Si)	2	314.62	1.44			383.45	1.61	403.96	1.72	218.95	1.08
	Abundance (Ab)	1	534.68	2.45			14.37	0.06	114.39	0.49	696.09	3.43
	Si x Ab	2	–				–		–		397.67	1.96
	Residual	26	218.07				238.08		234.23		203.13	

Analyses were done with permutational ANCOVA using Euclidean distances [48] testing correlations between percentage cover of *Ulva* and abundances of four common grazer species. Data used were densities per boulder, as well as numbers per boulder unstandardised according to boulder size. Eliminated interaction terms (*P* > 0.25) are denoted by “–”; *n* = 10.

*P < 0.05.

## Discussion

Seldom are soft-bodied algae such as *Ulva* spp. described from high levels of intertidal shores, where desiccation stress is extreme [50]. In the upper-shore they can survive when protected in rock-pools [51] or humid crevice environments [52], or when rain occurs [53]. This study has described, for the first time to my knowledge, *Ulva* spp. apparently shifting its niche and colonising the upper-shore via provision of biogenic habitat by tubeworms. The pattern was striking, with no instances of any *Ulva* being detected on any co-occurring boulders, while up to 61% occurred on boulders with tubeworms. This effect appeared to be driven by facilitation from serpulimorph polychaetes, and/or their empty tubes, made available following disturbance. Facilitation theory states that the habitat heterogeneity provided by early-successional colonists is greatly important for development of late-successional species diversity [17]; if facilitation is confirmed as the mechanism acting on *Ulva* in this high-shore boulder system, it would show how this habitat heterogeneity can be provided not only by new colonists shortly after disturbance, but also immediately after disturbance, if the disturbance event causes existing biogenic habitat produced in other habitats to become an available resource.

The structural complexity of serpulimorph tubes can provide biogenic habitat [54] and increase levels of species diversity [34]. Other tubeworms (*Diopatra cuprea*) can greatly facilitate algae, including *Ulva*, in soft-sediment habitats by providing stable substrata [55]. Similarly, the presence of the barnacle *Balanus improvisus* in the Baltic Sea can increase recruitment and growth of ephemeral algae [56]. These examples are in low-shore or subtidal habitats, and the current study did not test mechanisms causing associations of worm tubes and algae in the high-shore. Serpulimorph tubes are white, a colour that reduces *Ulva* recruitment [57], so other factors here are likely acting against effects of colour. Some possible mechanisms that might promote *Ulva* recruitment, and which could be tested in further studies, are water retention and heat stress. Mobile intertidal species can retain more moisture when aggregated [58, 59] (but see [60]) and clumping of sessile species can increase localised humidity and reduce heat-stress [61]. Similarly, aggregations of worm tubes here often appeared to visibly increase surface moisture compared to co-occurring boulders without worm tubes (personal observation). Complex substratum topographies can also increase larval settlement of many species [62, 63], and substratum irregularities of similar scale to serpulimorph tubes can increase recruitment of *Ulva* [64]. Finally, it is possible that some of the serpulimorph tubes were still living, and their excretory products were acting as a nitrogen source, effectively fertilising the *Ulva* [65–67]. For example, this was the mechanism thought to be promoting growth of ephemeral algae in the presence of *B*. *improvisus* in the Baltic Sea [56].

The patterns described here suggest that grazing is likely another important factor in this system. Grazing pressure on opportunistic algae in the intertidal is generally strong when *Cellana tramoserica* [68] and *Nerita atramentosa* are present [69], but species such as these can have their grazing pressure reduced by disturbance, such as from shifting sand [9]. Here, associations between grazer assemblages and cover of *Ulva* only occurred consistently *within* surfaces of boulders with serpulimorph tubes present, suggesting the association was caused by provision of biogenic habitat following disturbance, rather than the disturbance event itself.

There was never any *Ulva* on boulders without serpulimorph tubes, but there were many boulders with tubes that also did not have much *Ulva* and these had greater abundances of *N*. *atramentosa*, which is a particularly strongly-interacting grazer [69]. Positive associations have been found between abundances of this snail and uneven surfaces [35], contrasting effects of uneven surfaces for other grazers such as *C*. *tramoscerica* [70]. The boulders with large abundances of *Ulva* may have been recently disturbed and colonised by the alga, but not yet colonised by *N*. *atramentosa*. For example, Robles [9] found that ephemeral algae were the first species to colonise intertidal rock surfaces disturbed by sand movement in California. Early-colonising crabs subsequently arrived and began grazing activity, but it was not until slow-colonising gastropod grazers arrived that the disturbance-induced algal blooms were suppressed [9]. The results from the current experiment do not, however, indicate at which stage grazers colonised each boulder (i.e. before or after the appearance of *Ulva*) and grazing pressure is one of many models that may explain negative correlations between *N*. *atramentosa* and *Ulva*.

Algae of the *Ulva* genus are considered “pioneers” [71] with colonisation occurring soon after disturbance [21, 72]. Resources such as light and space are normally provided by disturbance [1], and this study has increased our knowledge of disturbance-related resources by showing how biogenic habitat can also be provided. The inferences in this study were from mensurative experimentation, and further research is required to manipulatively test the causal mechanism(s) of the association, including the role, indicated here correlatively, of grazers. Tests of the generality of the patterns and processes over larger spatiotemporal scales would also be ideal. These dynamics may have important implications for the maintenance of species diversity through facilitation in high-shore intertidal systems where disturbance processes interact with provision of biogenic habitat.

## Supporting information

S1 File Raw data. (XLS)

## References

[1] Sousa WP. The role of disturbance in natural communities. Annual Review of Ecology and Systematics. 1984; 15: 353–391.

[2] Canham CD, Denslow JS, Platt WJ, Runkle JR, Spies TA, White PS. Light regimes beneath closed canopies and tree-fall gaps in temperate and tropical forests. Canadian Journal of Forest Research. 1990; 20: 620–631.

[3] Guo D, Mou P, Jones RH, Mitchell RJ. Temporal changes in spatial patterns of soil moisture following disturbance: an experimental approach. Journal of Ecology. 2002; 90: 338–347.

[4] Robertson GP, Crum JR, Ellis BG. The spatial variability of soil resources following long-term disturbance. Oecologia; 1993 96: 451–456. doi: 10.1007/BF00320501 28312450 10.1007/BF00320501

[5] Dayton PK. Competition, disturbance, and community organization: the provision and subsequent utilization of space in a rocky intertidal community. Ecological Monographs. 1971; 41: 351–389.

[6] Marion GM, Moreno JM, Oechel WC. Fire severity, ash deposition, and clipping effects on soil nutrients in chaparral. Soil Science Society of America Journal. 1991; 55: 235–240.

[7] Tilman D. The resource-ratio hypothesis of plant succession. The American Naturalist. 1985; 125: 827–852.

[8] Sousa WP. Experimental investigations of disturbance and ecological succession in a rocky intertidal algal community. Ecological Monographs. 1979; 49: 227–254.

[9] Robles C. Disturbance and predation in an assemblage of herbivorous *Diptera* and algae on rocky shores. Oecologia. 1982; 54: 23–31. doi: 10.1007/BF00541103 28310987 10.1007/BF00541103

[10] Connell JH, Slatyer RO. Mechanisms of succession in natural communities and their role in community stability and organization. The American Naturalist. 1977; 111: 1119–1144.

[11] Soliveres S, Smit C, Maestre FT. Moving forward on facilitation research: response to changing environments and effects on the diversity, functioning and evolution of plant communities. Biological Reviews. 2014; 90: 297–313. doi: 10.1111/brv.12110 24774563 10.1111/brv.12110 PMC4407973

[12] Thomsen MS, Wernberg T, Altieri A, Tuya F, Gulbransen D, McGlathery KJ, Holmer M, Silliman BR. Habitat cascades: the conceptual context and global relevance of facilitation cascades via habitat formation and modification. Integrative and Comparative Biology. 2010; 50: 158–175. doi: 10.1093/icb/icq042 21558196 10.1093/icb/icq042

[13] McIntire EJB, Fajardo A. Facilitation as a ubiquitous driver of biodiversity. New Phytologist. 2013; 201: 403–416. doi: 10.1111/nph.12478 24102266 10.1111/nph.12478

[14] Bulleri F, Bruno JF, Silliman BR, Stachowicz JJ. Facilitation and the niche: implications for co-existence, range shifts and ecosystem functioning. Functional Ecology. 2016; 30: 70–78.

[15] Dethier MN. Disturbance and recovery in intertidal pools: maintenance of mosaic patterns. Ecological Monographs. 1984; 54: 99–118.

[16] Turner T. Facilitation as a successional mechanism in a rocky intertidal community. American Naturalist. 1983; 121: 729–738.

[17] Stachowicz JJ. Mutualism, facilitation, and the structure of ecological communities. BioScience. 2001; 51: 235–246.

[18] Farrell TM. Models and mechanisms of succession: an example from a rocky intertidal community. Ecological Monographs. 1991; 61: 95–113.

[19] Denny MW. Lift as a mechanism of patch initiation in mussel beds. Journal of Experimental Marine Biology and Ecology. 1987; 113: 231–245.

[20] Wernberg T, Connell SD. Physical disturbance and subtidal habitat structure on open rocky coasts: effects of wave exposure, extent and intensity. Journal of Sea Research. 2008; 59: 237–248.

[21] Sousa WP. Disturbance in marine intertidal boulder fields: the nonequilibrium maintenance of species diversity. Ecology. 1979; 60: 1225–1239.

[22] Shanks AL, Wright WG. Adding teeth to wave action: the destructive effects of wave-borne rocks on intertidal organisms. Oecologia. 1986; 69: 420–428. doi: 10.1007/BF00377065 28311345 10.1007/BF00377065

[23] Benedetti-Cecchi L, Pannacciulli F, Bulleri F, Moschella PS, Airoldi L, Relini G, Cinelli F. Predicting the consequences of anthropogenic disturbance: large-scale effects of loss of canopy algae on rocky shores. Marine Ecology Progress Series. 2001; 214: 137–150.

[24] Foster MS. Organization of macroalgal assemblages in the Northeast Pacific: the assumption of homogeneity and the illusion of generality. Hydrobiologia. 1990; 192: 21–33.

[25] Underwood AJ, Petraitis PS. Structure of intertidal assemblages in different locations: how can local processes be compared? In: Ricklefs RE, Schluter D, editors. Species Diversity in Ecological Communities: Historical and Geographical Perspectives. University of Chigaco Press Chicago; 1993 pp. 38–51.

[26] Underwood AJ, Chapman MG, Connell SD. Observations in ecology: you can’t make progress on processes without understanding the patterns. Journal of Experimental Marine Biology and Ecology. 2000; 250: 97–115. 10969165 10.1016/s0022-0981(00)00181-7

[27] Addessi L. Human Disturbance and Long‐Term Changes on a Rocky Intertidal Community. Ecological Applications. 1994; 4: 786–797.

[28] Liversage K, Kotta J. Disturbance-related patterns in unstable rocky benthic habitats of the north-eastern Baltic coast. Proceedings of the Estonian Academy of Sciences. 2015; 64: 53–61.

[29] McGuinness KA. Disturbance and organisms on boulders I. Patterns in the environment and the community. Oecologia. 1987; 71: 409–419. doi: 10.1007/BF00378715 28312989 10.1007/BF00378715

[30] James RJ, Underwood AJ. Influence of colour of substratum on recruitment of spirorbid tubeworms to different types of intertidal boulders. Journal of Experimental Marine Biology and Ecology. 1994; 181: 105–115.

[31] Chapman MG. The use of sandstone blocks to test hypotheses about colonization of intertidal boulders. Journal of the Marine Biological Association of the United Kingdom. 2003; 83: 415–423.

[32] Liversage K. Ecology of Cryptic Habitats Under Intertidal Boulders. PhD Thesis, the University of Sydney. 2012.

[33] Knight-Jones EW, Knight-Jones P. Four new species of *Eisothistos* (Anthuridea: Isopoda) from tubes of Spirorbidae (Serpuloidea: Polychaeta). Journal of Natural History. 2002; 36: 1397–1419.

[34] Moore CG, Saunders GR, Harries DB. The status and ecology of reefs of *Serpula vermicularis* L. (Polychaeta: Serpulidae) in Scotland. Aquatic Conservation: Marine and Freshwater Ecosystems. 1998; 8: 645–656.

[35] McGuinness KA, Underwood AJ. Habitat structure and the nature of communities on intertidal boulders. Journal of Experimental Marine Biology and Ecology. 1986; 104: 97–123.

[36] Hurlbert SH. Pseudoreplication and the design of ecological field experiments. Ecological Monographs. 1984; 54: 187–211.

[37] Kupriyanova EK, Nishi E, Ten Hove HA, Rzhavsky AV. Life-history patterns in serpulimorph polychaetes: ecological and evolutionary perspectives. Oceanography and Marine Biology: An Annual Review. 2001; 39: 1–101.

[38] Liversage K, Janetzki N, Benkendorff K. Associations of benthic fauna with different rock types, and evidence of changing effects during succession. Marine Ecology Progress Series. 2014; 505: 131–143.

[39] Chilton NB, Bull CM. Influence of predation by a crab on the distribution of the size-groups of three intertidal gastropods in South Australia. Marine Biology. 1984; 83: 163–169.

[40] Bulleri F, Russell BD, Connell SD. Context-dependency in the effects of nutrient loading and consumers on the availability of space in marine rocky environments. PLoS One. 2012; 7: e33825 doi: 10.1371/journal.pone.0033825 22457792 10.1371/journal.pone.0033825 PMC3311547

[41] Liversage K, Benkendorff K. A preliminary investigation of diversity, abundance, and distributional patterns of chitons in intertidal boulder fields of differing rock type in South Australia. Molluscan Research. 2013; 33: 24–33.

[42] Chapman MG. Patterns of spatial and temporal variation of macrofauna under boulders in a sheltered boulder field. Austral Ecology. 2002; 27: 211–228.

[43] Liversage K. Habitat associations of a rare South Australian sea star (*Parvulastra parvivipara*) and a co-occurring chiton (*Ischnochiton variegatus*): implications for conservation. Pacific Conservation Biology. 2015; 21: 234–242.

[44] McGuinness KA. Species-area relations of communities on intertidal boulders: testing the null hypothesis. Journal of Biogeography. 1984; 11: 439–456.

[45] Underwood AJ. Experiments in Ecology: Their Logical Design and Interpretation Using Analysis of Variance. Cambridge: Cambridge University Press; 1997.

[46] Anderson MJ, Walsh DCI. PERMANOVA, ANOSIM, and the Mantel test in the face of heterogeneous dispersions: What null hypothesis are you testing? Ecological Monographs. 2013; 83: 557–574.

[47] Anderson MJ. A new method for non-parametric multivariate analysis of variance. Austral Ecology. 2001; 26: 32–46.

[48] Anderson MJ, Gorley RN. Clarke KR. PERMANOVA+ for PRIMER: Guide to Software and Statistical Methods. Plymouth: PRIMER-E; 2008.

[49] Underwood AJ. Experiments in ecology and management: their logics, functions and interpretations. Australian Journal of Ecology. 1990; 15: 365–389.

[50] Davison IR, Pearson GA. Stress tolerance in intertidal seaweeds. Journal of Phycology. 1996; 32: 197–211.

[51] Underwood AJ. The effects of grazing by gastropods and physical factors on the upper limits of distribution of intertidal macroalgae. Oecologia. 1980; 46: 201–213. doi: 10.1007/BF00540127 28309674 10.1007/BF00540127

[52] Kensler CB. Desiccation resistance of untertidal crevice species as a factor in their zonation. Journal of Animal Ecology. 1967; 36: 391–406.

[53] Underwood AJ, Jernakoff P. The effects of tidal height, wave-exposure, seasonality and rock-pools on grazing and the distribution of intertidal macroalgae in New South Wales. Journal of Experimental Marine Biology and Ecology. 1984; 75: 71–96.

[54] Wright JT, Gribben PE, Latzel S. Native ecosystem engineer facilitates recruitment of invasive crab and native invertebrates. Biological Invasions. 2016; 18: 3163–3173.

[55] Thomsen MS, McGlathery KJ. Facilitation of macroalgae by the sedimentary tube forming polychaete *Diopatra cuprea*. Estuarine, Coastal and Shelf Science. 2005; 62: 63–73.

[56] Kotta J, Kotta I, Simm M, Lankov A, Lauringson V, Põllumäe A, Ojaveer H. Ecological consequences of biological invasions: three invertebrate case studies in the north-eastern Baltic Sea. Helgoland Marine Research. 2006; 60: 106–112.

[57] Swain G, Herpe S, Ralston E, Tribou M. Short-term testing of antifouling surfaces: the importance of colour. Biofouling. 2006; 22: 425–429. doi: 10.1080/08927010601037163 17178575 10.1080/08927010601037163

[58] Chapperon C, Le Bris C, Seuront L. Thermally mediated body temperature, water content and aggregation behaviour in the intertidal gastropod *Nerita atramentosa*. Ecological Research. 2013; 28: 407–416.

[59] Chapman MG, Underwood AJ. Influences of tidal conditions, temperature and desiccation on patterns of aggregation of the high-shore periwinkle, *Littorina unifasciata*, in New South Wales, Australia. Journal of Experimental Marine Biology and Ecology. 1996; 196: 213–237.

[60] Coleman RA. Limpet aggregation does not alter desiccation in the limpet *Cellana tramoserica*. Journal of Experimental Marine Biology and Ecology. 2010; 386: 113–118.

[61] Nicastro KR, Zardi GI, McQuaid CD, Pearson GA, Serrão EA. Love thy neighbour: group properties of gaping behaviour in mussel aggregations. PloS ONE. 2012; 7: e47382 doi: 10.1371/journal.pone.0047382 23091620 10.1371/journal.pone.0047382 PMC3472978

[62] Callow ME, Jennings AR, Brennan AB, Seegert CE, Gibson A, Wilson L, Feinberg A, Baney R, Callow JA. Microtopographic cues for settlement of zoospores of the green fouling alga *Enteromorpha*. Biofouling. 2002; 18: 229–236.

[63] Schumacher JF, Carman ML, Estes TG, Feinberg AW, Wilson LH, Callow ME, Callow JA, Finlay JA, Brennan AB. Engineered antifouling microtopographies–effect of feature size, geometry, and roughness on settlement of zoospores of the green alga *Ulva*. Biofouling. 2007; 23: 55–62. doi: 10.1080/08927010601136957 17453729 10.1080/08927010601136957

[64] Harlin MM, Lindbergh JM. Selection of substrata by seaweeds: Optimal surface relief. Marine Biology. 1977; 40: 33–40.

[65] Williamson JE, Rees TAV. Nutritional interaction in an alga-barnacle association. Oecologia. 1994; 99: 16–20. doi: 10.1007/BF00317078 28313943 10.1007/BF00317078

[66] Taylor R, Rees TAV. Excretory products of mobile epifauna as a nitrogen source for seaweeds. Limnology and Oceanography. 1998; 43: 600–606.

[67] Bracken MES, Nielsen KJ. Diversity of intertidal macroalgae increases with nitrogen loading by invertebrates. Ecology. 2004; 85: 2828–2836.

[68] Underwood AJ, Jernakoff P. Effects of interactions between algae and grazing gastropods on the structure of a low-shore intertidal algal community. Oecologia. 1981; 48: 221–233. doi: 10.1007/BF00347968 28309804 10.1007/BF00347968

[69] Underwood AJ. An experimental evaluation of competition between three species of intertidal prosobranch gastropods. Oecologia. 1978; 33: 185–202. doi: 10.1007/BF00344847 28309163 10.1007/BF00344847

[70] Underwood AJ, Denley EJ, Moran MJ. Experimental analyses of the structure and dynamics of mid-shore rocky intertidal communities in New South Wales. Oecologia. 1983; 56: 202–219. doi: 10.1007/BF00379692 28310196 10.1007/BF00379692

[71] Littler MM. Morphological form and photosynthetic performances of marine macroalgae: tests of a functional/form hypothesis. Botanica Marina. 1980; 23: 161–166.

[72] Airoldi L, Bulleri F. Anthropogenic disturbance can determine the magnitude of opportunistic species responses on marine urban infrastructures. PLoS One. 2011; 6: e22985 doi: 10.1371/journal.pone.0022985 21826224 10.1371/journal.pone.0022985 PMC3149642

