# Correction: First evidence of biogenic habitat from tubeworms providing a near-absolute habitat requirement for high-intertidal *Ulva* macroalgae

**DOI:** 10.1371/journal.pone.0192579

**Published:** 2018-02-05

**Authors:** Kiran Liversage

There is text missing from Fig 4. Please see the correct Fig 4 here.

**Fig 4:**
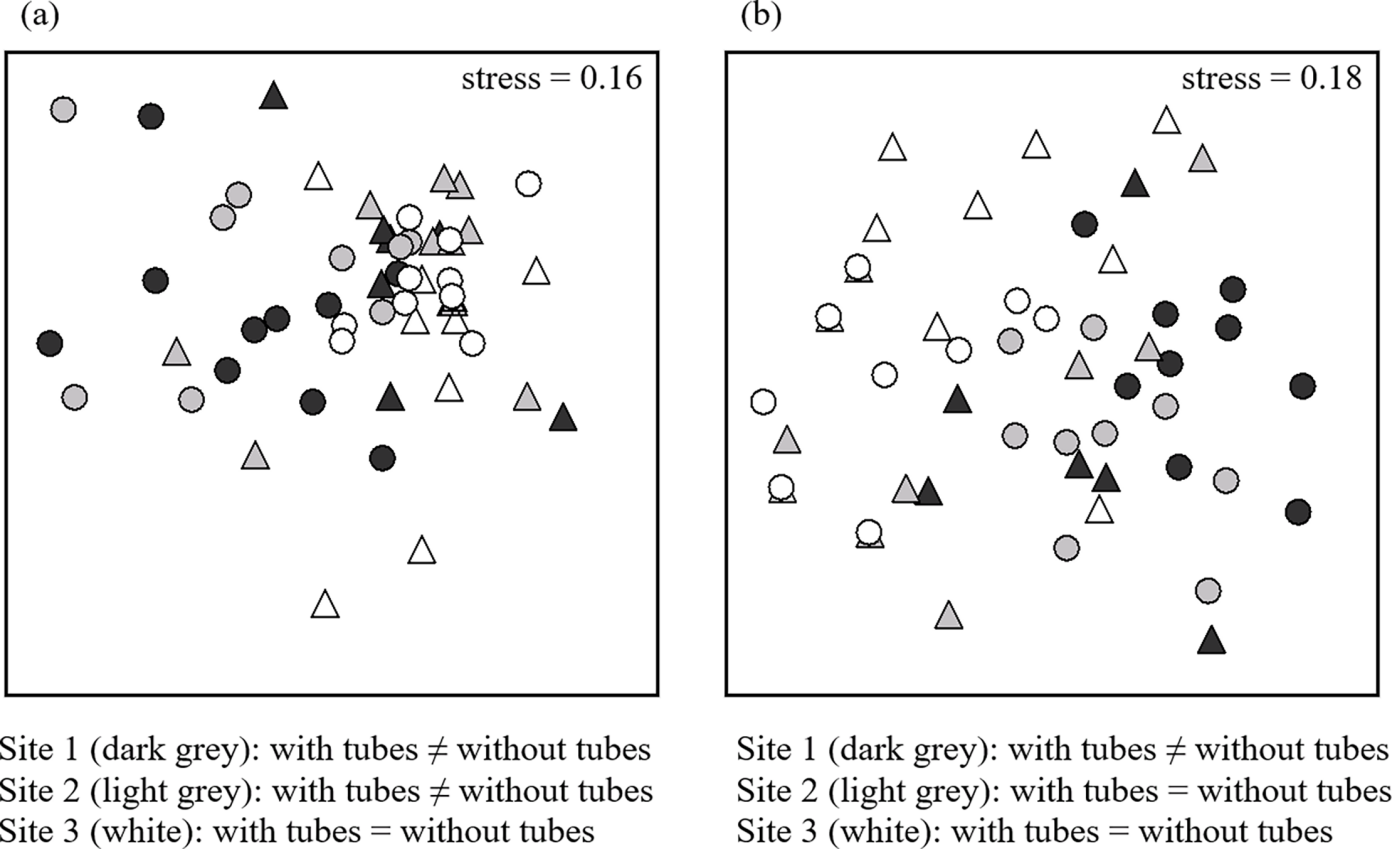
Associations of grazer assemblages with worm-tubes vary according to random sites. nMDS plots of (a) densities, and (b) numbers per boulder, of mobile assemblages (grazers) on boulders with serpulimorph tubes (triangle) and without (circle). Dark grey symbols are from Site 1, light grey from Site 2, and white from Site 3. Results from PERMANOVA pairwise tests are shown under the plots; *n* = 10.

There is an error in the caption for Fig 6. Please see the correct Fig 6 caption here.

**Fig 6:**
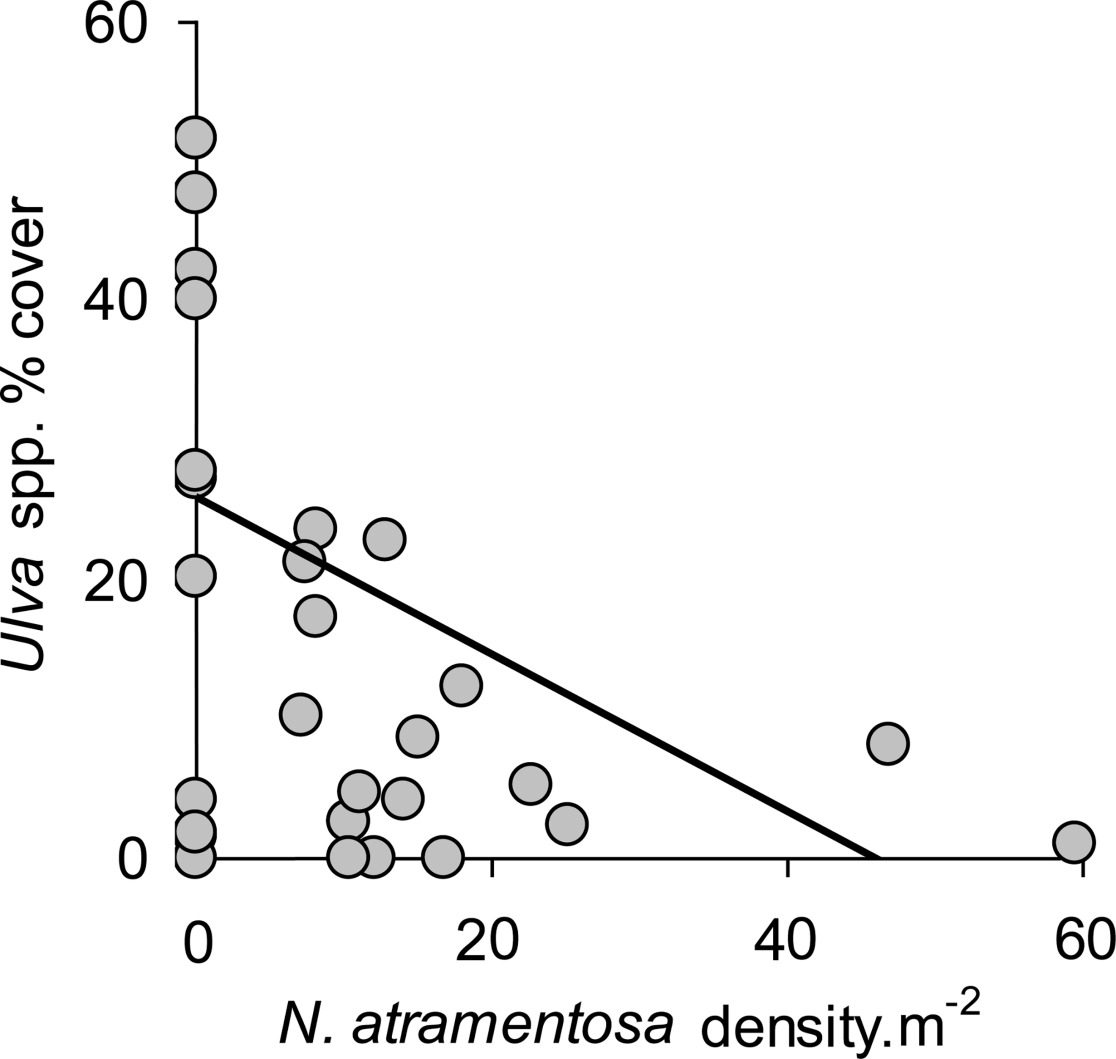
Density of the grazer *Nerita atramentosa* is negatively correlated with *Ulva* algae cover. Correlation between density of the grazer *N*. *atramentosa* and cover of *Ulva* (arcsine transformed; [49]) on boulders which had serpulimorph tubes. Linear regression was used to visually represent the result from the permutational ANCOVA. Data were pooled from three random sites; *n* = 10.
